# Radiobiological Determination of Dose Escalation and Normal Tissue Toxicity in Definitive Chemoradiation Therapy for Esophageal Cancer^☆^

**DOI:** 10.1016/j.ijrobp.2014.06.028

**Published:** 2014-10-01

**Authors:** Samantha Warren, Mike Partridge, Rhys Carrington, Chris Hurt, Thomas Crosby, Maria A. Hawkins

**Affiliations:** ∗ Department of Oncology, Gray Institute of Radiation Oncology and Biology, University of Oxford, Oxford, United Kingdom; † Velindre Cancer Centre, Velindre Hospital, Cardiff, United Kingdom; ‡ Wales Cancer Trials Unit, School of Medicine, Heath Park, Cardiff, United Kingdom

## Abstract

**Purpose:**

This study investigated the trade-off in tumor coverage and organ-at-risk sparing when applying dose escalation for concurrent chemoradiation therapy (CRT) of mid-esophageal cancer, using radiobiological modeling to estimate local control and normal tissue toxicity.

**Methods and Materials:**

Twenty-one patients with mid-esophageal cancer were selected from the SCOPE1 database (International Standard Randomised Controlled Trials number 47718479), with a mean planning target volume (PTV) of 327 cm^3^. A boost volume, PTV2 (GTV + 0.5 cm margin), was created. Radiobiological modeling of tumor control probability (TCP) estimated the dose required for a clinically significant (+20%) increase in local control as 62.5 Gy/25 fractions. A RapidArc (RA) plan with a simultaneously integrated boost (SIB) to PTV2 (RA_62.5_) was compared to a standard dose plan of 50 Gy/25 fractions (RA_50_). Dose-volume metrics and estimates of normal tissue complication probability (NTCP) for heart and lungs were compared.

**Results:**

Clinically acceptable dose escalation was feasible for 16 of 21 patients, with significant gains (>18%) in tumor control from 38.2% (RA_50_) to 56.3% (RA_62.5_), and only a small increase in predicted toxicity: median heart NTCP 4.4% (RA_50_) versus 5.6% (RA_62.5_) *P*<.001 and median lung NTCP 6.5% (RA_50_) versus 7.5% (RA_62.5_) *P*<.001.

**Conclusions:**

Dose escalation to the GTV to improve local control is possible when overlap between PTV and organ-at-risk (<8% heart volume and <2.5% lung volume overlap for this study) generates only negligible increase in lung or heart toxicity. These predictions from radiobiological modeling should be tested in future clinical trials.

**Summary**

Radiobiological modeling suggests that dose escalation to the gross tumor volume in esophageal cancer has the potential to produce significant gains in tumor control with only a minor increase in lung or heart toxicity for the majority of patients. The relationship between tumor response and normal tissue toxicity during dose escalation should be carefully validated in clinical trials.

## Introduction

Chemoradiation therapy (CRT) is becoming established as an effective treatment for locally advanced esophageal cancer, as a neoadjuvant strategy for operable adenocarcinomas or as a definitive treatment when there is a high risk of surgical morbidity and mortality (1). Long-term survival for operable squamous cell carcinomas treated with definitive CRT (dCRT) is comparable to surgery alone (2). Although CRT is more effective than either radiation therapy or chemotherapy alone 3, 4, locoregional control rates with the standard radiation therapy dose of ≈50 Gy are still low, and >75% of failures occur within the gross tumor volume (GTV) 5, 6.

A correlation between higher dose and improved tumor control and survival was described by Zhang et al (7) when patients were divided into low-dose (≤51 Gy) and high-dose (>51 Gy) groups. Further evidence of a radiation dose response has been found by a systematic review (8), which looked at rates of pathological complete response (pCR) in preoperative CRT. Fitting the data to a radiobiological model suggested that increasing the radiation dose prescription from the standard ≈50 Gy could result in significant improvement in tumor control probability (TCP).

Conversely, data from a phase 3 clinical trial, Radiation Therapy Oncology Group (RTOG) 9405, investigating the use of higher radiation dose (64.8 Gy) versus standard dose (50.4 Gy) found no improvement in survival or local control, and a relatively high comorbidity in the high-dose arm (9). However, the radiation therapy treatment technique at this time was based on 2D planning (using relatively large treatment fields) and a sequential dose boost regimen. Occurrence of several deaths in the high-dose arm before reaching 50.4 Gy has limited further investigation of dose escalation in spite of general understanding of 2D planning insufficiency.

The role of radiation therapy dose escalation in improving outcomes for definitive CRT has been recently identified as one of the priorities for research in esophageal cancer in the United Kingdom (10). Nevertheless, the optimal combination of radiation therapy and chemotherapy doses needs to be carefully established in order to improve locoregional control.

Long-term toxicities, particularly to the cardiorespiratory system have been described following dCRT for esophageal cancer. Acute toxicity related to substructures of the heart has been studied specifically, for example, pericardial effusion (onset within 6 months) 11, 12, 13. These studies suggest a higher mean pericardial dose is associated with increased risk of pericardial effusion. Left ventricular mean dose (14) has also been found to be related to acute cardiac impairment observed by MRI for patients undergoing CRT for esophageal cancer. Radiobiological models of long-term cardiac toxicity have also been presented using dose to whole heart (15).

Radiation-induced lung toxicity is important, not just as a result of the proximity of lung tissue to the esophageal target volume (16) but also as newer radiation therapy techniques such as IMRT or volumetric arc, generate a low dose bath to a large region of lung tissue. Recent studies of lung cancer also indicate that radiation-induced lung toxicity may be related to either pre-existing cardiac co-morbidity (17) or to concomitant irradiation of the heart (18). Although the biological mechanism is as yet unclear, there is a statistically significant association between risk of radiation pneumonitis and dose-volume variables for both heart and lung for these patients.

This study had 2 aims: (*1*) to estimate the level of dose escalation required for a clinically significant increase in tumor control using radiobiological modeling across a *group* of representative patients, and to examine whether this dose could be safely delivered by calculating normal tissue complication probability (NTCP) for heart and lungs; and (*2*) to evaluate the feasibility of a clinical trial using dose escalation for esophageal cancer patients treated with definitive CRT by comparing results for *individual* patients.

## Methods and Materials

A subset of 21 mid-thoracic esophageal cancer patients (10% of the total radiation therapy group) was selected from the SCOPE1 database (ISRCTN 47718479). This subset had a range of planning target volumes of 140-591 cm^3^ (Table 1) and a mean PTV (327 cm^3^) consistent with the entire original cohort (mean 334 cm^3^). Trial-derived gross tumor volumes (GTV) were used, and visual assessment of normal tissue contours was undertaken. The SCOPE protocol standard margins were re-applied without modification to generate the clinical target volume (CTV) and planning target volume (PTV1) (19). Several additional volumes were contoured: PTV2 (boost volume) created by adding a 0.5 cm margin in all directions to the GTV, and a pericardium structure comprised of a 1 cm rind inside (12) the whole heart outline.

**Table 1:** Patient target volume characteristics and overlap with heart and lung structures in order of increasing PTV1 size∗

Patient	Volume (cm^3^)			% Volume overlap	
	GTV	PTV2	PTV1	Lung in PTV1	Heart in PTV1
1	4.7	18.4	140.1	0.5	1.0
2	4.1	16.7	146.8	1.0	4.9
3	7.2	28.8	195.2	0.2	6.7
4	10.5	32.7	195.6	0.5	5.5
5	8.8	29.0	205.2	0.4	7.6
6	15.3	43.4	218.6	0.4	3.8
**7**	**12.1**	**43.1**	**233.4**	**0.2**	**9.6**
8	16.5	48.9	239.0	0.8	5.2
9	27.7	66.1	297.7	2.0	8.6
10	30.2	71.6	301.1	1.3	6.3
**11**	**23.3**	**66.1**	**311.7**	**0.8**	**16.5**
12	29.3	76.8	329.8	2.0	4.6
13	37.2	92.7	356.1	0.8	4.6
14	45.1	101.2	374.9	0.5	5.5
15	52.3	111.0	405.2	1.9	4.9
**16**	**43.9**	**105.3**	**408.8**	**2.5**	**8.2**
**17**	**46.0**	**109.8**	**434.2**	**1.4**	**11.3**
18	35.4	94.1	443.1	1.5	3.6
19	65.5	140.3	453.6	1.7	2.6
20	95.4	175.2	544.9	1.8	5.0
**21**	**104.4**	**199.5**	**590.8**	**3.2**	**8.5**

*Abbreviations:* GTV = gross tumor volume; PTV = planning target volume.

∗ Patients for whom dose escalation was unsuccessful are marked in boldface type.

Radiobiological modeling of TCP to determine the level of dose escalation to the GTV was carried out using the parameters from Geh et al (8). This is a multivariate logisitic regression model fitted to data from 26 CRT trials for preoperative esophageal cancer. Analysis was based on the protocol-prescribed doses of radiation therapy and chemotherapy (5-fluorouracil and cisplatin) to predict the TCP (TCP Geh) of pCR, and included total dose, dose per fraction and duration among the fitting parameters. The values of the covariates and coefficients used were those listed in the original paper (8).

$$\mathrm{TCP}\left(z\right)=\frac{\text{exp}\left(z\right)}{1+\text{exp}\left(z\right)}$$

where

$$z=a_{0}+a_{1}\;\mathrm{total RT dose}+a_{2}\;\mathrm{total RT dose}\times \mathrm{dose per fraction}+a_{3}\;\mathrm{duration}+a_{4}\;\mathrm{age}+a_{5}\;5\mathrm{FU dose}+a_{6}\;\mathrm{cisplatin dose}$$

A clinically significant 20% increase in local control requires 67.0 Gy, but this would considerably lengthen the total treatment time if delivered in 2-Gy fractions. We therefore calculated the equivalent dose to be delivered in 25 fractions (2.5 Gy per fraction) as 62.5 Gy, maintaining the same duration (33 days) as the standard plans of 50 Gy.

Treatment planning was performed using Eclipse, version 10 (Varian, Palo Alto, CA). RapidArc (RA) plans were created using 2 arcs of 360°, clockwise and counter-clockwise, with a collimator rotation of ±10°. Dose calculation was performed using the AAA algorithm using a 2.5-mm grid. A standard plan (RA_50_) was created (dose prescription 50 Gy/25 fractions to PTV1) and compared to a plan with an additional simultaneously integrated boost (SIB) of 62.5 Gy to PTV2 (RA_62.5_). Dose constraints are listed in Table 2, and additional dose-volume metrics were recorded for pericardium (mean dose, V_30Gy_, V_45Gy_) and lung V_13Gy_.

**Table 2:** Dose constraints used in treatment planning for RA_50_ and RA_62.5_ radiation therapy plans

Dose-volume	Constraint
PTV1 (50 Gy)	V_95%_ (47.5 Gy) > 95% D_max_ (0.1 cc) < 107% (53.5 Gy)
PTV2 (GTV + 0.5 cm) (62.5 Gy)	V_95%_ (59.375 Gy) > 95% D_max_ (0.1 cc) < 107% (66.875 Gy)
Lung	Mean dose < 20 Gy V_20Gy_ < 25%
Heart	Mean dose < 25 Gy V_30Gy_ < 45%
CordPRV	D_max_ (0.1 cc) < 40 Gy (45 Gy permitted)

*Abbreviations:* CordPRV = Cord planning organ at risk volume; Dmax = maximum dose; RA_50_ = RapidArc plan to 50 Gy; RA_62.5_ = RapidArc plan with boost to 62.5 Gy; V_30Gy_ = volume receiving 30 Gy.

The differential dose-volume histogram (DVH) for the GTV was exported for each dose plan. TCP calculations were performed bin-wise using the Webb-Nahum model (20) with parameters from reference (21) (TCP Bedford) and also the Geh et al (8) model (TCP Geh). NTCP modeling was carried out for heart, using the whole-heart volume model of Gagliardi et al (15), for the pericardium (12), and for lung using the model parameters from De Jaeger et al (22), which predict radiation pneumonitis (RP) of grade 2 or higher (symptoms requiring steroids). In addition, we applied a combined heart and lung irradiation model (18) to calculate predicted risk of RP in these patients, where the dose to the “hottest” 10% of the heart volume (D_10H_), and the mean lung dose (MLD) in Gy are used to calculate NTCP

$$\mathrm{NTCP}=\frac{1}{1+\text{exp}\left(-x\right)}$$

where

$$x=0.0234\times D_{10_{H}}+0.0649\times \mathrm{MLD}-3.5$$

As the values of dose-volume metrics, TCP and NTCP across all patients were not normally distributed for all sets of data (Shapiro-Wilk test), we used the Wilcoxon signed rank test to compare RA_50_ versus RA_62.5_ plans for N=21 patients. Data were analyzed using SPSS, version 20.0.0 (IBM), and results are reported as median (range) values, with *Z* and *P* listed in Table 3.

**Table 3:** Comparison of dose-volume metrics, TCP, and NTCP values∗

Dose-volume	Plan		
	Median RA_50_ (range)	Median RA_62.5_ (range)	RA_62.5_-RA_50_ (Wilcoxon signed-rank test)
PTV1			
V_95%_	98.3 (95.3-100)	96.7 (90.5-98.7)	*Z* = 2.63, *P*<.001
PTV2			
V_95%_		97.2 (93.9-99.3)	
TCP Geh et al (8) (%)	38.2 (37.6-39.6)	56.3 (55.1-57.2)	*Z* = 4.02, *P*<.001
TCP Bedford et al (21) (%)	40.3 (39.2-43.2)	71.7 (70.1-72.9)	
Lung			
Mean dose (Gy)	12.6 (8.4-18.2)	13.3 (8.4-17.9)	*Z* = 3.77, *P*<.001
V_13Gy_ (%)	42.2 (19.7-68.6)	46.4 (19.7-68.8)	*Z* = 3.82, *P*<.001
V_20Gy_ (%)	12.6 (5.9-29.9)	15.9 (5.8-29.1)	*Z* = 3.74, *P*<.001
NTCP (%) De Jaeger et al (22)	6.5 (3.9-12.9)	7.5 (3.9-12.6)	*Z* = 3.81, *P*<.001
NTCP (%) Huang et al (18)	14.2 (10.9-22.0)	15.5 (10.8-21.5)	*Z* = 3.84, *P*<.001
Heart			
Mean dose (Gy)	20.4 (13.2-27.9)	20.3 (12.9-30.0)	*Z* = 1.41, *P*=.16
V_30Gy_ (%)	16.6 (10.3-33.1)	18.0 (10.5-38.1)	*Z* = 2.38, *P*=.02
NTCP (%) Gagliardi et al (15)	4.4 (2.3-10.4)	5.6 (2.5-14.8)	*Z* = 3.98, *P*<.001
Pericardium (1cm inner)			
Mean dose (Gy)	20.8 (13.8-27.9)	22.0 (14.2-29.6)	*Z* = 2.86, *P*=.004
V_30Gy_ (%)	19.6 (12.1-33.4)	20.7 (12.3-35.0)	*Z* = 3.29, *P*=.001
V_45Gy_ (%)	10.9 (6.5-23.2)	10.7 (6.0-23.7)	*Z* = 1.14, *P*=.25
CordPRV			
Dmax 0.1 cc (Gy)	34.9	36.1	*Z* = 2.54, *P*=.01

*Abbreviations:* CordPRV = Cord planning organ at risk volume; Dmax = maximum dose; NTCP = normal tissue complication probability; PTV = planning target volume; RA_50_ = RapidArc boost to 50 Gy; RA_62.5_ = RapidArc plan with boost to 62.5 Gy; TCP = tumor control probability; V_45Gy_ = volume receiving 45 Gy.

∗ Although the median values for dose to organs at risk for RA_50_ and RA_62.5_ plans are statistically different (*P*<.05), except for heart mean dose, the differences in median dose across all patients are not clinically significant.

## Results

Dose boosting to 62.5 Gy with adequate target dose coverage was possible for 20 out of 21 patients. For patient 21 (largest PTV, with 18.6% of PTV1 in lung) only 90.5% coverage by the 95% isodose contour for PTV1 was obtained. For all other patients the minimum V95% was 94.7% (RA_62.5_) versus 95.3% (RA_50_). For 4 additional patients (7, 11, 16, 17) it was not possible to increase dose to the GTV without exceeding other dose constraints. Failure was due to lung V_20Gy_ > 25% (patient 16) and mean heart dose > 25 Gy (patients 7, 11, and 17). The dose escalation strategy was therefore successful for 16/21 (76%) of patients.

**Fig. 1:**
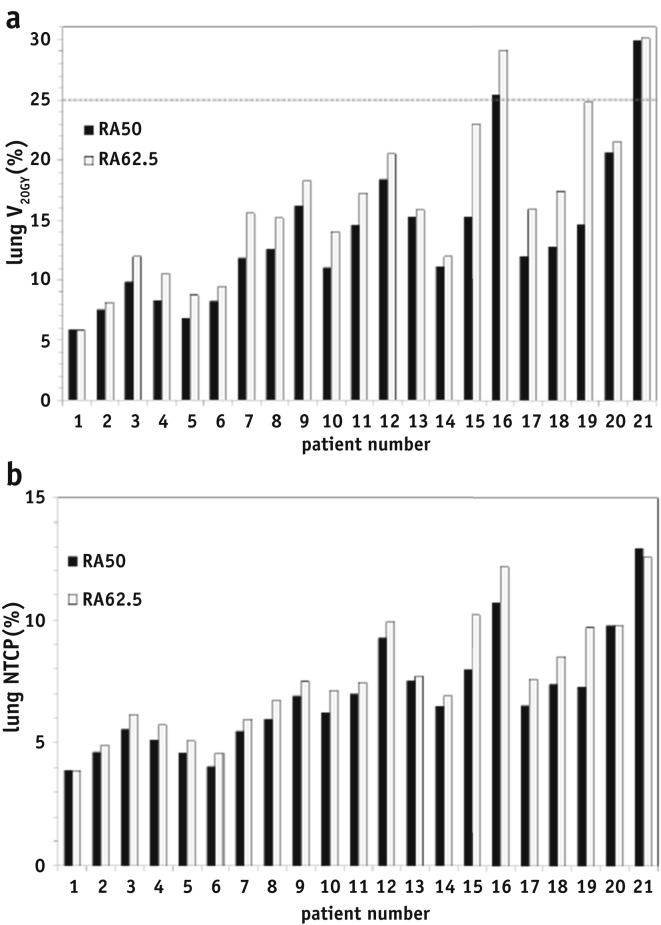
(a) Lung V20Gy values for each patient in order of increasing PTV size for plans RA_50_ (black bars) and RA_62.5_ (gray bars). The dose-volume constraint of 25% is shown as a dashed line. (b) Lung NTCP was calculated using the model parameters of De Jaeger for plans RA_50_ (black bars) and RA_62.5_ (gray bars) for each patient. NTCP = normal tissue complication probability; PTV = planning target volume; RA_50_ = RapidArc plan to 50 Gy; RA_62.5_ = RapidArc plan with boost to 62.5 Gy; V20Gy = volume receiving 20 Gy.

The calculated TCP (Table 3) increased by 18% (from 38.2% to 56.3%) on average for the Geh model, and 31% (from 40.3% to 71.7%) on average for the Bedford model. Figure 1 shows patients listed in order of increasing PTV1 size and illustrates the general trend of increased irradiation of the lung with increasing target volume size, although mean lung dose is <20 Gy for all patients, and the average increase with dose escalation is less than 1 Gy. The lung V_20Gy_ constraint was exceeded only for patients 16 and 21 (RA_50_ and RA_62.5_ plans), who have the highest percentage of lung overlap in PTV1 of 2.5% and 3.2% respectively (Table 1). Lung V_13Gy_ shows an average increase of 4.2% (Table 3) for the higher dose plans.

**Fig. 2:**
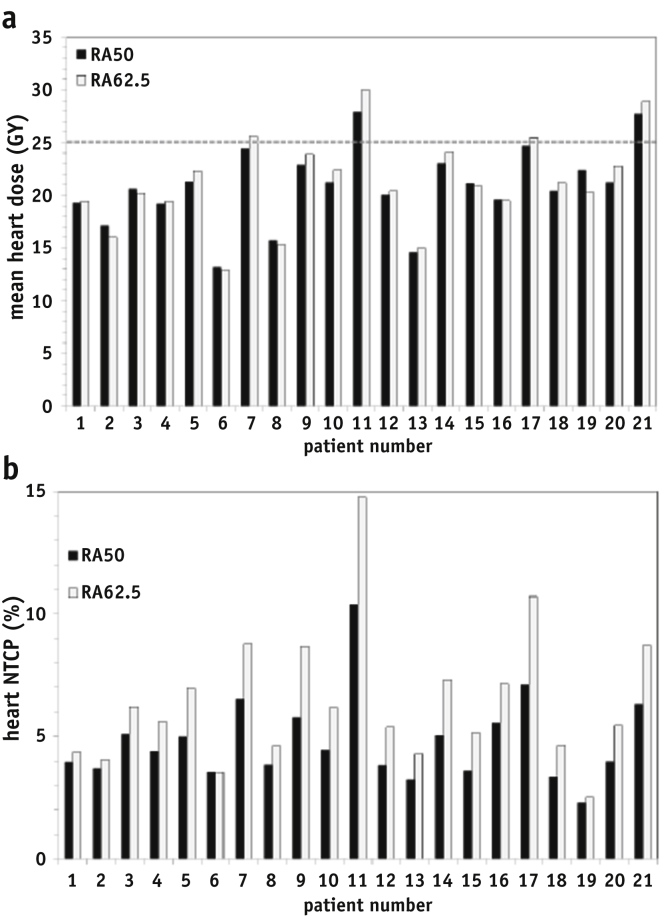
(a) Mean heart dose (Gy) for each patient for plans using RA_50_ (black bars) and RA_62.5_ (gray bars). The dose-volume constraint of 25 Gy is shown as a dashed line; (b) predicted heart NTCP for each patient using the whole heart contour and the NTCP model from Gagliardi et al (15). NTCP = normal tissue complication probability; RA_50_ = RapidArc plan to 50 Gy; RA_62.5_ = RapidArc plan with boost to 62.5 Gy.

The SIB plans (RA_62.5_) result in an increase on average of less than 1 Gy in mean dose to heart and pericardium. Irradiation of the heart was not directly dependent on tumor size (Table 1), but on the overlap with the PTV1 volume (Fig. 2a). Patients 7, 11, and 17 have the highest % heart overlap in PTV1 (9.6%, 16.5%, and 11.3% respectively) and mean heart dose was exceeded when the boost dose was applied (plan RA_62.5_). For patient 11, the mean heart dose constraint could not be met for either plan, due to the GTV abutting the heart contour for this patient. Heart V_30Gy_ was well below the 45% limit for all patients. Mean dose to the pericardium inner rind (12) was less than the recommended 27.1 Gy dose constraint for all patients except patient 11 (27.7 Gy) and patient 21 (27.9 Gy).

Heart mortality predicted using the criteria by Gagliardi et al (15) showed a statistically significant increase of (on average) 1.2% with increased dose to the GTV (Table 3), though the predicted increase in heart mortality for some individual patients can be larger (Fig. 2b) due to overlap of heart with PTV1 (listed in Table 1). NTCP modeling for pericardial effusion (12) applied to the pericardial structure predicted a risk of pericarditis of zero for all patients.

For lung, the changes in NTCP (22) vary from patient to patient (Fig. 1b). On average, risk of grade 2 (or higher) radiation pneumonitis increased from 6.5% (RA_50_) to 7.5% (RA_62.5_) *P*<.001 (Table 2). Patients 20 and 21 with large PTV show no increase in predicted lung NTCP with higher GTV dose, due to the trade-off with lower target coverage. Using the combined heart and lung irradiation model for radiation pneumonitis (23), the predicted risks of RP are greater than for the lung only model, though the increase in predicted RP with dose escalation is on average < 1% (median NTCP: 14.2% [RA_50_] versus 15.5% [RA_62.5_] *P*<.001).

## Discussion

Our analysis of the dose-volume metrics and radiobiological modeling of dose escalation for esophageal cancer suggests that a significant increase (+18%) in tumor control can potentially be achieved with only a modest increase in the risk of cardiac and lung toxicities for 76% of patients in our study of a subset of the SCOPE clinical trial database. Other authors have also shown that IMRT may reduce dose to critical structures such as heart and coronary arteries, when compared to conformal radiation therapy techniques 24, 25, but our study is the first to use radiobiological modeling to estimate both the level of dose escalation, and to predict heart and lung toxicities arising from this increase in dose. Patient outcomes and dose response will be monitored in the proposed randomized clinical trial investigating dose escalation for esophageal cancer (SCOPE-2).

The results of our analysis are, of course, dependent on the choice of the dose-volume and radiobiological parameters and models used, and we have therefore used a range of models and values from the literature. We have also shown that the relative comparison of 2 fractionation schemes is valid using 2 independent TCP models, although the absolute values of predicted TCP may be different, depending on the model parameters. By comparing fraction schedules with the same duration, we have also limited any error due to clonogenic repopulation rates and uncertainty in kick-off time. Both TCP models assume an identical dose response for adenocarcinomas and squamous cell esophageal cancers, and our analysis assumed a homogeneous distribution of clonogens within the GTV. If the 0.5 cm margins applied to create the boost volume around the GTV are insufficient to ensure adequate dose coverage in the presence of tumor motion, this may reduce the predicted increase in TCP, such that these values represent the upper limit of the benefits conferred by dose escalation.

Recent data from cine-MRI suggest a margin of 10.7 ± 0.4 mm in the cranial-caudal direction might be required to cover 95% of tumor movement (26), but this would imply an increase in dose to a significant portion of esophagus located immediately superior and inferior to the primary tumor, which may be a dose limiting factor in these patients. A margin of 0.5 cm was chosen in order to limit the volume of “healthy” esophagus irradiated and to generate a gradual drop-off in dose around the GTV. Data available in the literature concerning the dose-volume relationship for esophageal toxicity are from lung cancer patients 27, 28, and it is not clear if the dose limits would be applicable to patients with esophageal tumors. The results of a clinical trial using hypofractionation in non-small cell lung cancer (NSCLC) observed several grade 4 and 5 toxicities to central structures when doses were increased to 75 Gy and above but suggested 63.25 Gy/25 fractions should be well-tolerated in terms of both acute and late toxicity (29), which would suggest our proposed dose prescription of 62.5 Gy/25 fractions may be acceptable. We have in addition used stricter dose-volume constraints for plan optimization than specified in the original SCOPE protocol, but careful monitoring of esophageal toxicity during and after CRT will be necessary for the safe application of any dose escalation scheme.

For patients with larger PTVs with significant overlap in lung (>540 cm^3^ and 18% overlap in our patient subset), dose escalation using arc therapy may not be possible without a significant increase in dose delivered to surrounding lung. Hybrid static IMRT/RA irradiation techniques might provide better lung sparing for these patients (30). Also, the CTVs created in our study to encompass regions of microscopic tumor spread, were generated using isotropic geometrical margins. A further improvement would be to delineate the CTV for each patient on each slice of the CT scan to respect known anatomical boundaries, and thereby reduce the amount of normal tissue irradiated.

For patients where the heart overlaps the PTV (>8.5% heart in PTV overlap for this subset) dose escalation would exceed recommended cardiac dose constraints, even using advanced treatment planning and delivery techniques. Dose to the pericardium might be considered more relevant for modeling of acute toxicity (pericardial effusion) than dose to the whole heart, although our data suggests that for all patients, dose to pericardium is well within the various dose-volume constraints recommended in the literature 11, 12, 13. This may be due to the use of IMRT/RA, which delivers significantly lower dose to heart than previous 2D or 3D conformal radiation therapy techniques. We have therefore used the whole heart to model cardiac mortality, although as a long-term effect (5-10 years), this may be less clinically relevant for esophageal cancer patients where median survival is around 25 months (31).

Acute radiation-induced lung toxicity may therefore be more important for these patients and our data suggest that even with higher dose to the tumor, the increase in risk of radiation pneumonitis is small, and might be considered acceptable for patients with limited organ-at-risk overlap inside the PTV (<8% heart volume and <2.5% lung volume overlap for this study), given the predicted gains in tumor control. Concomitant irradiation of the heart or a pre-existing cardiac pathology have recently been identified as risk factors in radiation pneumonitis (18), and this should be assessed for each patient during planning.

## Conclusions

Radiobiological modeling suggests that dose escalation to the GTV in esophageal cancer has the potential to produce significant gains in tumor control with only a minor increase in lung or heart toxicity for the majority of patients. The relationship between tumor response and normal tissue toxicity during dose escalation should be carefully validated in clinical trials.
